# Methods for the Identification of Outliers and Their Influence on Exposure Assessment in Agricultural Pesticide Applicators: A Proposed Approach and Validation Using Biological Monitoring

**DOI:** 10.3390/toxics7030037

**Published:** 2019-07-12

**Authors:** Stefan Mandić-Rajčević, Claudio Colosio

**Affiliations:** Department of Health Sciences, University of Milan and International Centre for Rural Health of the Saints Paolo and Carlo Hospital, 20142 Milan, Italy

**Keywords:** pesticides, environmental monitoring, biological monitoring, mancozeb, ethylene thiourea, statistical method

## Abstract

The “patch” approach for skin exposure assessment can easily be combined with biological monitoring in real-life pesticide studies. Nevertheless, this approach is sensitive to outliers, with values markedly deviating from other members of the sample, which can result in a gross overestimation of exposure. This study aimed at developing methods for outlier identification and validating them while using biological monitoring. Twenty-seven workers applying mancozeb in Italian vineyards participated in this study. Their skin exposure was estimated while using the patch methodology, while ethylene-thiourea (ETU) was measured in the 24-h post-exposure urine as a biomarker of exposure. The outliers were detected using methods that were based on the multiplication of the median, the median absolute deviation, and boxplots. The detection rate varied between 2.3% and 17.3%. The estimated median skin exposure of 3.2 μg was reduced to 1.2 μg when the modified Z score was used. The highest reduction in the skin exposure was above 54 μg. The use of the modified Z score for outlier detection resulted in an increase in the correlation coefficient between the skin exposure and the urine ETU levels from 0.46 to 0.71, which suggested the validity of the approach. Future studies should standardize and improve the methods for pesticide exposure and risk assessment.

## 1. Introduction

The most commonly used methods for pesticide exposure assessment in field studies are biological and environmental monitoring. Biological monitoring uses biological samples (e.g., urine) to evaluate exposure while considering all routes and, in some cases, it is even possible to reconstruct the dose that can then be used for risk assessment through comparison with the Acceptable Operator Exposure Level (AOEL) [1,2]. The lack of biomarkers, validated protocols for specimen collection, and biological exposure limits are some of the downsides of biomonitoring for risk assessment [3]. On the other hand, environmental monitoring directly measures the exposure, while considering each route separately. Skin, or the dermal route, is regarded as the most critical route of exposure in open field pesticide application, contributing to more than 90% of the total dose [4,5]. Therefore, correctly estimating skin exposure is fundamental for exposure assessment of pesticide use in agriculture.

The “whole-body” and “patch” approaches, falling under “surrogate skin methods”, represent the backbone of skin exposure assessment [6]. They have been described in detail in official guidelines [7] and extensively used in field studies [8,9,10,11]. The main disadvantage of the whole body approach is that it changes the regular working routine of the worker by imposing an additional layer of protection to the worker, which renders the study experimental as opposed to “real-life”. Additionally, the parallel use of biological monitoring becomes impractical, as the whole-body sampling collects the total skin dose, leaving nothing to be absorbed. The patch method, on the other hand, involves the use of several smaller-surface samplers that measure the exposure of various body regions. Only a small percentage of the body surface is “protected” by the patches, which makes the parallel use of biomonitoring feasible, while not changing the standard routine of the worker [12]. However, patches represent samples of body region exposures, which leads to a higher probability of measuring values below the limit of detection or extremely high values, which might lead to errors during the extrapolation from the patch to the body region it represents. The importance of correctly estimating “real” exposure in epidemiological studies has been previously underlined [13], and the same issue has been discussed more recently in various attempts to achieve a more realistic risk assessment in the exposure to mixtures [14,15]. The approaches to exposure and risk assessment that consider “real-life scenarios” are receiving more attention, but the main problem is represented by the variability of “real-life” data, which has yet to be addressed [2,16]. The simulation of exposures amounting from various combinations of stressors and the development of novel methodological approaches to estimate real-life exposures is necessary [17,18,19].

Values that are lower than the limit of detection can be replaced or imputed, and studies have shown that their treatment does not significantly change the overall exposure assessment [20]. Outliers, or extremely high values, which are defined as observations numerically distant from the rest of the data, can reduce data quality and cause erroneous judgment. Due to extrapolation, which is done by multiplying the value measured on a patch by the surface of the region it represents, higher-than-expected contamination of a single patch may result in a gross overestimation of exposure. In the field of environmental health, outlier identification and management have been a hot topic in soil, air, and wastewater data. Methods, such as boxplots, spatial autocorrelation (Moran’s I value), variogram clouds, the probability theory, and difference maps, have been found to be useful in this context [21,22,23]. Nonetheless, only two studies on pesticide exposure mention outlier detection and exclusion, basing the decision on professional judgment [24,25]. Nevertheless, some of the above-mentioned methods, such as boxplots and the probability distribution, could be applied to pesticide exposure of workers. Biological monitoring can be considered for the confirmation of exposure in studies employing both environmental and biological monitoring, which should correlate well with correctly assessed skin exposure while using the patch approach.

The aim of this study was to propose and test methods for outlier identification, quantify their influence on the overall exposure assessment, and validate the approach while using biological monitoring in a group of agricultural workers applying Mancozeb in Italian vineyards.

## 2. Materials and Methods

In 2011, a large, real-life, pesticide exposure study was conducted in the Mantova and Pavia provinces of the Region of Lombardy (Northern Italy). Enterprises applying a well-known ethylene-bisdithiocarbamate pesticide, mancozeb, were identified with the help of local authorities and while using sales data, and invited to participate in the study. The Ethics Committee of the San Paolo University Hospital of Milan (Italy) approved the study. Before the beginning of the study, employers and employees were informed about the methods and aims of the study. All of the participants signed the informed consent form. The study included three points of data collection: (a) a previously validated Data Collection Sheet that was specifically designed for pesticide exposure and risk studies; (b) skin exposure to mancozeb while using the “patch” methodology; and, (c) excretion of ethylenethiourea, the primary metabolite of mancozeb, in the 24-h post-exposure urine samples of the study group. The inclusion criteria were no occupational exposure to ethylene-bisdithiocarbamates in the previous 15 days, which excluded some of the workers who were monitored for several working days. A total of 27 workers from the original study sample were included in the present study, with each applying mancozeb during one work day.

### 2.1. Data Collection Sheet

The Data Collection Sheet that was used in this study has been published previously [18]. It was based on a literature review regarding the main determinants of pesticide exposure to be considered for the creation of a simple tool for estimate pesticide exposure and the related risk in typical agriculture scenarios [2,26]. It contains all of the questions necessary for identifying the agricultural enterprise addressed (usually a small-size enterprise) and its main profile. The company name, type of crop, treated surface, and number of workers were the main data that were collected. The primary information that was collected from the worker(s) involved has been gender, age, anthropometric data, dominant hand (left/right), as well as data regarding their experience in pesticide application. As for working activities, the data collection sheet addressed the work days considered, with information regarding the performed tasks, their duration, the quantities of active substance used, and the personal protection that was adopted. All the data regarding the above-mentioned characteristics of the work and their influence on the exposure levels have been previously published and they are not discussed in the present study [12].

### 2.2. Assessment of Skin Exposure

Exposure of the workers’ skin (hands excluded) was measured according to the Organization for Economic Co-operation and Development (OECD) guideline [7], with some modifications [10,12]. In particular, six rectangular 0.01 m^2^ pads that were made of Whatman n°1 filter paper (Prodotti Gianni, Milan) were placed on the skin of the workers. These pads aimed at estimating the actual skin exposure, defined as the amount of the active substance reaching the skin, thus available for absorption. The six locations were: chest, back, left forearm, right forearm, left thigh, and right thigh. The total body surface of each study subject was calculated while using the Mosteller approach [27]. The percentages of the body surface represented by each pad were calculated while using the “rule of nines”, which was usually used for the estimation of the injured skin surface in burn victims’ [28]. The original study also included the evaluation of the exposure on clothes (potential skin exposure), as well as hands’ exposure. This data has been published previously and it will not be discussed in the present study.

### 2.3. Assessment of Urine ETU Excretion

Mancozeb’s primary metabolite, ethylene-bis-thiourea (ETU), was measured in the 24-h post-exposure urine samples collected in large hospital urine containers. The collection of post-exposure urine started at the end of the application and lasted for the next 24-h. Containers were stored closed at +4 °C until they were transported to the laboratory for sample preparation and analysis.

### 2.4. Sample Preparation and Analysis

All of the samples were analyzed using liquid chromatography-mass spectrometry, namely the Acquity UPLC system (Waters, Milford, MA, USA) coupled with a triple quadrupole Waters TQD mass spectrometer. Free ETU was determined in line with the previously published methods [29]. Quantification was done while using the TQD detector with an ESI interface in positive ion mode (ESI+). The MRM acquisition used to quantify the free ETU was: m/z 103 → 44 (CV 36, CE 16); for internal standard 2H4-ETU quantification was obtained in SIR: m/z 107 (CV35). UPLC separation was performed on a Waters UPLC HSS T3 1.8 µm (2.1 × 100 mm) column that was kept at 28 °C, by gradient elution with a mixture containing a variable proportion of water and methanol, delivered at a flow rate of 0.4 mL/min. The retention time of ETU and its internal standard was 1.3 min. A detailed description of the sample preparation, analysis, limits of detection, and quantification, as well as quality assessment and quality control, is available in our previously published paper [19].

### 2.5. Exposure Assessment

The absolute amount that was found in each pad was calculated in μg from the original concentrations (μg/L) found in individual samples. The skin exposure for each region that was represented by the pads was extrapolated, having in mind the estimated surface of each body region and the amount measured on the pads. All of the regional exposures were summed to amount for the total skin exposure (see Equation (1)):

(1) $$Skin\;exposure=\sum_{Pad=1}^{6}Pad\left(\frac{\mu g}{dm^{2}}\right)\times Body\;region\;represented\;\left(dm^{2}\right),$$

### 2.6. Outlier Detection and Statistical Analyses

Data management, processing, and statistical analyses were performed while using the R Language and Environment for Statistical Computing with additional packages [30]. Three groups of methods were implemented in R to detect the outliers in our sample of workers. The methods were based on multiplication, median absolute deviation, and boxplots.

#### 2.6.1. Multiplication

This group of methods was based on a simple multiplication of the median value of the levels measured on the pads of each worker

(a) Median ×10

A pad was flagged as an outlier in case its value was 10 times higher than the median value of pads for the worker. In the text, this method is denoted by Med10.

(b) Median ×100

A pad was flagged as an outlier in case its value was 100 times higher than the median value of pads for the worker. This method is denoted by Med100 In the text.

#### 2.6.2. Modified Z Score

This score was developed as a standardized score to measure how much a particular score differs from a typical score. Contrary to the classic Z score, which is used in measurements with a normal distribution, the modified Z score approximates the difference of a score from the median value. Iglewicz and Hoaglin (1993) recommended it as:

(2) $$Mi=\frac{0.6745\times \left(x_{i}-\tilde{x}\right)}{MAD}$$

where: *MAD* denotes the median absolute deviation and $\tilde{x}$ denotes the median value. The authors recommended that an absolute modified Z-score value that was greater than 3.5 be labeled as a potential outlier [31]. This method is denoted by ZMad in the text.

#### 2.6.3. Boxplot

This method is commonly used in statistical software to indicate outliers.

(a) Q3 + 1.5 *IQR*

A pad was flagged as an outlier in the case its value was higher than the third quartile value plus 1.5 times the interquartile range (IQR). In the text, this method is denoted by IQR15.

(b) Q3 + 2.5 *IQR*

A pad was flagged as an outlier in case its value was higher than the third quartile value plus 2.5 times the interquartile range. This method is denoted by IQR25 in the text.

In the case of value flagged as an outlier, it was replaced by the amount that was found on the contralateral side of the body of the same worker. Each of the proposed methods was compared with the non-treated results in tables and figures. Spearman’s rank-order correlation was used to determine the association between the estimated skin exposure (without and using the proposed methods) and the levels of ETU excreted in 24-h urine, and the Spearman’s rank correlation coefficient (Spearman’s rho, ρs) is reported in the text and figures.

Categorical data were presented as the number of observations in each category and the corresponding percentage. Numerical data were first graphically analyzed, and the normality of their distribution was checked while using the Shapiro–Wilk test. Numerical data were presented as means and standard deviations in the case the Shapiro–Wilk test confirmed the normal distribution. Otherwise, numerical data are presented as median values with the first and third quartile values (Q1–Q3).

## 3. Results

This study included 27 male agricultural workers while only applying the fungicide mancozeb on the workday without any previous exposure. Seven agricultural workers used Open tractors, while 20 used Closed and filtered tractors. The median height and weight of the workers were 176 cm and 86 kg, respectively, which resulted in the median body surface area of 2.04 m^2^. Their median experience as pesticide applicators was 18 years, with a minimum of 4, and a maximum of 38 years doing this work. Details regarding the work that was performed during the work day, equipment at their disposal, personal protective devices and their use in various phases of work, and their influence on the exposure levels are presented elsewhere [12].

**Figure 1:**
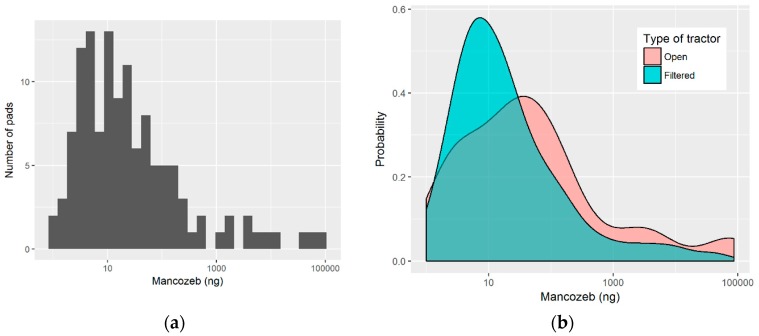
Distribution of the measured pad exposure: (**a**) Histogram of exposure levels measured in individual pads; (**b**) Probability density function of the measured exposures depending on the tractor type.

**Figure 2:**
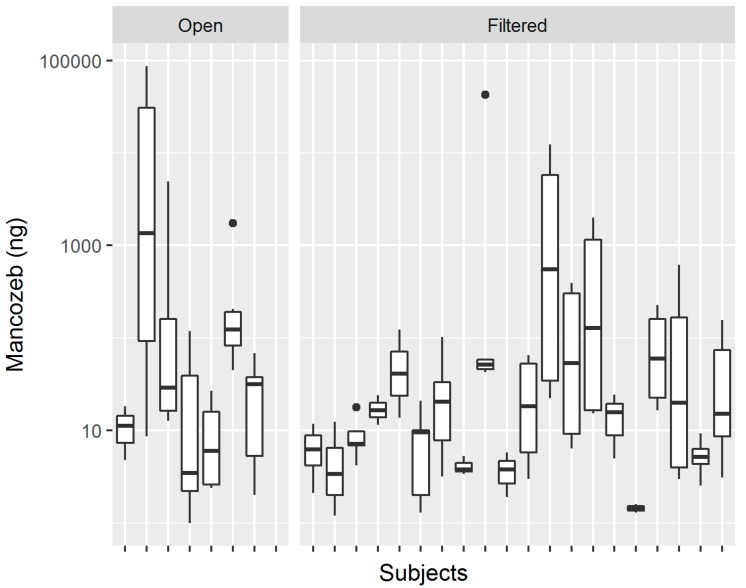
Boxplots of pad exposure by subjects divided by tractor type.

The skin exposure of each worker was measured using six patches, which were positioned on their chest, back, left and right forearm, and left and right thigh. Figure 1 shows the distribution of the measured values of mancozeb in the skin pads of workers, with Panel A showing the highest number of pads grouping on the left around the 10 ng value, while Panel B shows the difference in the distribution of pad exposure levels between the open and filtered tractor workers. Table 1 presents a summary of the levels of Mancozeb measured on the pads. The most exposed pads were those that were located on the right arm, with a median exposure of 60 nanograms, while the least exposed pad was that on the chest, with a median exposure of 6.9 nanograms. It is interesting to note that the workers using closed and filtered tractors (denoted by “Filtered” in Tables) had a higher median exposure on their back and chest when compared to the workers using open tractors, with values of 13.9 and 9.2 ng, as compared to 4.8 and 2.4, respectively. On the other hand, workers using open tractors had higher exposures that were measured on their arm and leg pads, which were commonly one order of magnitude greater than that found in workers while using closed and filtered tractors. Figure 2 shows the boxplots of pad exposure for each worker, divided by the type of tractor. The *y*-axis is log transformed to capture the wide range of values, and the boxplots demonstrate the intra-worker variability between the measured values on the pads, as well as the inter-worker, and even the inter-group variability in the measured pad values.

Five methods were proposed for the detection of outliers (see Section 2). The detection rate varied from 2.38% (four pads, Med100 method) up to 17.33% (26 pads, ZMad method). Table 2 shows the number and percentage of outliers that were detected while using the proposed method. A similar proportion of pads were flagged as outliers in both groups of workers, using the same method. Table 3 presents a summary of exposure measured on flagged pads. Pads that were flagged by the Med100 and IQR25 methods had the highest median values of 3440 and 615 ng, respectively. As expected, values that were flagged as outliers were higher in open, as opposed to closed and filtered tractors, up to an order of magnitude.

**Figure 3:**
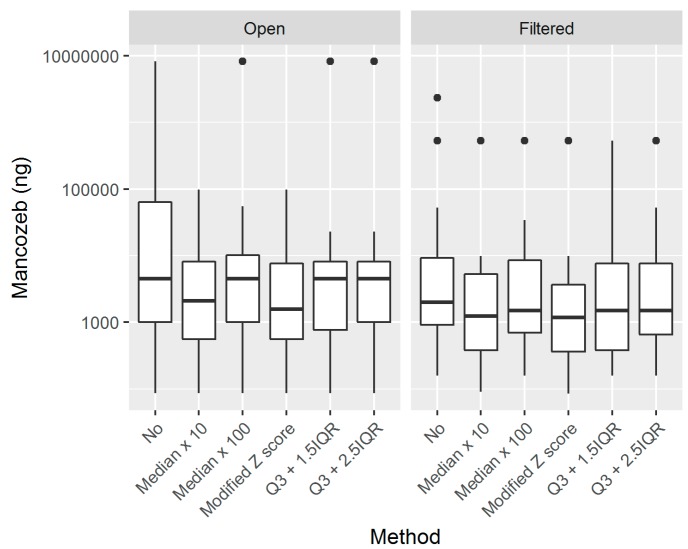
Boxplots of workers’ skin exposure without any outlier treatment (“No”) and while using the proposed methods, divided by tractor type.

The median skin exposure that was estimated without outlier detection was 3219 ng, with somewhat higher results in open tractor workers when compared to filtered tractor workers, namely, 4595 and 2066 ng, respectively. Table 4 shows the skin exposure estimates without and while using the proposed methods, as well as the biological monitoring results. The biomonitoring results presented are the 24-h post-exposure ETU levels, 24-h post-exposure ETU levels corrected for creatinine, and the difference between the 24-h pre- and post-exposure ETU levels. The use of the modified Z score method resulted in the lowest median estimated skin exposure of 1188 ng, and reduced the median estimated skin exposure of the open and filtered workers by around 50%, arriving to 1904 and 1188 ng, respectively. The application of the proposed methods for outlier detection resulted in an important reduction of the estimated (extrapolated) individual and group exposure. Table 5 summarizes the reductions that resulted from the use of the proposed methods, in total and divided by tractor type. The highest median reduction in skin exposure estimates of 54,768 ng was seen when the Med100 method was used, while the lowest median reduction was seen when the IQR15 method was used. Higher median reduction values were found in open, as compared to closed, and filtered tractors. Figure 3 shows the boxplots of the estimated skin exposures in open and filtered tractor workers without any outlier detection (denoted by “No” in the figure) and while using the five proposed methods. The application of outlier detection methods resulted in the lowering of the median estimated exposure, as well as the variability of estimated exposure (denoted by the size of the boxplots) in both of the groups. The modified Z score method resulted in the highest reduction of the median estimated skin exposure in both groups.

Finally, Spearman correlation coefficients were calculated between the exposure estimates (without and using the proposed method for outlier detection) and biological monitoring results to validate the need for outlier detection, as well as to compare the adequacy of the proposed methods, represented by the measurements of ETU. Table 6 presents the Spearman correlation coefficients. The correlation coefficient between the skin exposure that was estimated without outlier detection and the 24-h post-exposure urine ETU levels was 0.46. Using any of the proposed outlier detection methods resulted in higher correlation coefficients, which indicated a need for outlier detection. The least improvement in the correlation coefficients was seen when the Med100 and IQR (both 1.5 and 2.5) methods were used, while a somewhat more significant improvement was seen when the Med10 method for outlier detection was used. The highest correlation of 0.71 was found between the skin exposure that was estimated while using the modified Z score outlier detection, and this was found consistently, regardless of the biological monitoring measure (i.e., 24-h post-exposure ETU levels, 24-h post-exposure ETU levels corrected for creatinine, or the difference between the 24-h pre- and post-exposure ETU levels).

## 4. Discussion

This paper evaluates the need for outlier detection for environmental (personal exposure) monitoring while using the “patch” methodology in agricultural workers applying pesticides. The advantage of the “patch” methodology is the possibility of keeping the original working conditions, and the use of biological monitoring in parallel to the environmental monitoring, but the extrapolation from patches to the whole body surface is a process that is prone to the influence of extreme values or outliers. We proposed five methods for outlier detection, two based on multiplying the median by 10 and 100, one based on the modified Z score and the median absolute deviation, and two based on boxplots. The proposed methods detected between a few, up to several dozen outlier values, which ultimately lead to a reduction in the skin exposure estimates. Biological monitoring results validated the proposed methods for outlier detection and the improvement of skin exposure estimates. The use of any of the proposed method resulted in an increase in the correlation coefficient denoting the association between the estimated skin exposure and excreted metabolite’s levels, underlining the importance of outlier detection.

The exposure that was measured on the pads ranged from several nanograms, up to several hundred, or even thousands of nanograms (see Table 1 and Figure 1). The high variability in the measured levels resulted from the variability that was commonly found in the agricultural application of pesticides, resulting from the differences in the equipment, hygienic practices, terrain, the use of personal protective equipment, and environmental conditions [32,33,34]. The back and the chest appear to be the most protected regions, while the arms (mostly right) and legs (also right) were the most exposed. Previous studies have shown the importance of the dominant hand in exposure [10]. Our research has shown a higher median exposure of the back and chest in closed and filtered tractor workers, when compared to open tractor workers, which is the first time to our knowledge that a study has shown this result. It is possible that, in the closed cabin and a more comfortable tractor, the worker is more pressed upon the back of his seat or is bent over the steering wheel. Combined with inadequate hygienic conditions inside, denoted by surface contamination [35], this could result in the increased exposure, which was already seen in closed tractor workers that were using protective gloves inside the cabin [12]. On the other hand, higher exposure was found on the arms and legs of open tractor workers. The high intra-worker variability (as exposure measured on the pads of a single worker), inter-worker variability, and inter-group variability (e.g., open vs. filtered tractor) indicate that the evaluation of potential outliers and their treatment should be based on the single subject. A measure considered an outlier for one worker likely might not represent an outlier for another.

Among the five proposed methods for outlier detection, the modified Z score flagged the highest number of pads (26, or 17%) as outliers, while the Med100 (median multiplied by 100) detected the lowest number (4, or 2.38%, see Table 2). This difference was to be expected, as the Med100 method is only based on the median value and an arbitrarily selected multiplicator of 100, which can only detect extreme deviations from the median, and it does not take into account the variability of data. The modified Z score, based on the median absolute deviation, takes into account the variability of data and it is similar to the use of the confidence interval or the Z-test in normally distributed measurements [31]. The median values that were flagged as outliers ranged between 122 and 3440 ng, with some more extreme values even one order of magnitude higher. When considering that these values would be multiplied by number as high as 40 to represent the surface of the body region, in the process of extrapolation (see Section 2), the exclusion of these extreme values naturally lead to important reductions in individual workers’ estimated skin exposure. Namely, the median reduction in the estimated (extrapolated) exposure ranged from 2231 up to 54,768 ng, or more than 54 μg (see Table 5). To put this value into context, the 3rd quartile level of exposure that was expected in open tractor workers without outlier detection was 65,793 ng, or 65 μg (see Table 4), while the median exposure that was found in open tractor workers without outlier detection was 4.5 μg, which is 10 times lower than the reduction that is achieved by outlier detection. Having in mind that the Acceptable Operator Exposure Level (AOEL) for mancozeb is 35 μg/kg of body weight, the detection of outliers could have important implications not only for exposure assessment, but also for risk assessment of exposed workers [36]. The use of proposed outlier detection methods resulted in the reduction of individual skin exposure estimates (see Table 4). From the estimated median skin exposure of 3219 ng in our workers, without outlier detection, the exposure was reduced to 1188 ng in the case of the modified Z score approach. A reduction in the variability was also evident (see Figure 3), which might facilitate the interpretation of the results. Defining the determinants of exposure and misclassification of exposure is one of the biggest problem in pesticide exposure and risk studies, as well as epidemiological studies dealing with pesticide health effects, as most of the existing surrogate measures of exposure are based on field data reports [2,37,38,39].

Finally, our approach was to use biological monitoring results, which indicated the “real” exposure that was absorbed by the workers, to validate the proposed methods, the need for their use and to propose the most relevant one. The correlation between our workers’ skin exposure estimates and post-exposure metabolite urine levels was 0.45 (see Table 6). The values of correlation coefficients, between 0.3 and 0.5, were found in previous studies that used environmental monitoring together with biological monitoring in tebuconazole and penconazole exposed workers [8,9,10]. In the present study, the use of any outlier detection method resulted in an increase in the correlation coefficient, which underlines the effectiveness of outlier detection. Among the five proposed approaches, the best results were achieved while using the modified Z score, which resulted in the correlation coefficient of 0.71 between the estimated skin exposure and 24-h post-exposure urine ETU levels. This validates the modified Z score approach for the detection of outliers in pesticide exposure studies while using the “patch” method.

Our previous studies have shown that quantifiable levels of ETU can be measured in the pre-exposure urine of agricultural pesticide applicators, and even in the Italian general population [12,20]. The main source of this “background” exposure is believed to be food and wine containing pesticide residues or their degradation products [40]. Nevertheless, the use of outlier detection was able to improve the correlation between the estimated exposure and the “difference between pre- and post-exposure urine ETU levels”, a measure that was intended to reduce the influence of background, rendering this measure of occupational exposure useful for future attempts of modelling or developing biological exposure limits.

Most of the limitations of this study are connected with the number of participants and the difficulty of measuring “real” exposure, which could then be compared to the “estimated” one. The number of participants in this study (28) can be considered to be above average for real-life pesticide exposure and risk studies, which usually include around 10 participants [4,5,10]. The lack of “real” exposure measurement is a problem that is yet to be solved, as all the methods for environmental and biological monitoring of pesticide exposure suffer from some drawbacks. A step forward in solving this problem would be proposing universally acceptable and validated protocols for pesticide field studies, including data collection, processing, extrapolation, and reporting of results, which would allow for the outcomes to be more generalizable and reusable. A data collection sheet that is based on the determinants of pesticide exposure has already been developed, as well as the method for also taking the duration of exposure in estimating the absorbed dose of an active substance into account [18,19]. Future studies might focus on the integrated use of the existing tools, as well as on the development of better approaches to improve pesticide exposure and risk assessment, possibly based on the parallel use of the “patch” and “whole-body” method, as well as the re-analysis of existing data.

## 5. Conclusions

Correctly estimating the skin exposure in pesticide-exposed workers is of crucial importance for risk assessment and absorbed dose assessment. The “patch” methodology requires the use of an outlier detection method to correctly identify the extreme values that might lead to an overestimate of exposure during the extrapolation process. The use of the modified Z score, based on the median absolute deviation, resulted in the identification of a large number of extreme values, which were treated to improve the accuracy of the skin exposure estimates. This method was validated by the use of biological monitoring results, which resulted in a higher correlation between the estimated skin exposure to mancozeb and the measured levels of mancozeb’s main metabolite, ETU. Future studies should implement this, or explore other more suitable methods for outlier detection, as well as continue to improve the process of exposure and risk assessment of pesticide use in agriculture.

## Figures and Tables

**Table 1:** Summary of exposure measured in nanograms on pads by tractor type and location of the pads.

Position	Type of Tractor					
	All Median (IQR)		Open Median (IQR)		Filtered Median (IQR)	
Back	7.51	(2.89–21.35)	2.40	(2.00–51.80)	9.23	(4.40–18.25)
Chest	6.90	(4.00–24.30)	4.80	(2.85–56.35)	13.90	(4.43–23.77)
Left arm	29.00	(11.50–166.00)	84.00	(23.65–183.25)	15.85	(6.87–151.00)
Right arm	60.00	(9.70–216.45)	119.00	(48.00–1128.35)	19.90	(9.44–145.75)
Left leg	7.90	(3.50–38.90)	16.30	(8.60–39.00)	4.60	(3.40–38.60)
Right leg	17.40	(5.93–38.47)	35.00	(29.10–45.00)	8.42	(5.35–28.25)

**Table 2:** Number of pads flagged as outliers by the proposed methods.

Method	Type of Tractor		
	All	Open	Filtered
Median × 10	20 (11.90%)	7 (14.58%)	13 (10.83%)
Median × 100	4 (2.38%)	1 (2.08%)	3 (2.50%)
Modified *Z* score	26 (17.33%)	8 (19.05%)	18 (16.67%)
Q3 + 1.5 IQR	11 (6.55%)	3 (6.25%)	8 (6.67%)
Q3 + 2.5 IQR	7 (4.17%)	2 (4.17%)	5 (4.17%)

**Table 3:** Summary of exposure levels in nanograms found on pads flagged by the proposed methods.

Method	Type of Tractor					
	All Median (IQR)		Open Median (IQR)		Filtered Median (IQR)	
Median × 10	251.95	(69.75–1802.88)	1737.00	(101.50–33,796.25)	228.00	(24.10–615.00)
Median × 100	3440.25	(1531.10–14,306.00)	4880.00	N/A	2000.50	(1061.70–22,292.25)
Modified Z score	159.25	(24.98–874.72)	949.25	(110.25–19,338.12)	139.95	(16.38–365.60)
Q3 + 1.5 IQR	122.90	(25.55–1868.75)	1737.00	(882.00–3308.50)	112.85	(22.55–961.38)
Q3 + 2.5 IQR	615.00	(112.85–3308.50)	3308.50	(2522.75–4094.25)	122.90	(102.80–615.00)

**Table 4:** Summary of skin exposure levels in nanograms depending on the outlier detection method and biological monitoring results.

	Method	Type of Tractor		
		All Median (IQR)	Open Median (IQR)	Filtered Median (IQR)
**Skin Exposure (ng)**				
	No outlier detection	3219.56 (939.73–14,393.91)	4595.57 (1006.27–65,793.22)	2066.12 (921.81–9521.81)
	Median × 10	1238.42 (439.58–5749.27)	3079.00 (558.78–10,068.12)	1238.42 (381.39–5332.08)
	Median × 100	2719.80 (822.17–8606.93)	4595.57 (1006.27–18,142.38)	1507.39 (703.23–8606.93)
	Modified Z score	1188.70 (391.59–5225.99)	1904.60 (558.78–9728.87)	1188.70 (362.61–3757.98)
	Q3 + 1.5 IQR	2672.78 (544.87–7640.25)	4595.57 (778.54–10,068.12)	1507.39 (381.99–7640.25)
	Q3 + 2.5 IQR	2672.78 (822.17–7640.25)	4595.57 (1006.27–10,068.12)	1507.39 (675.77–7640.25)
**Biological monitoring**				
	24-h post-exposure ETU levels (μg)	2.59 (1.29–7.45)	3.02 (1.38–11.53)	2.39 (1.29–4.78)
	24-h post-exposure ETU level corrected for creatinine (μg/g creat.)	2.07 (1.14–5.03)	3.02 (1.33–8.21)	1.95 (1.11–4.05)
	Difference between 24-h pre- and post-exposure ETU levels (μg)	1.53 (0.06–6.46)	1.83 (0.32–7.68)	1.17 (<0.01–3.93)

**Table 5:** Extent of reduction in extrapolated worker exposure in nanograms depending on the method used.

Method	Type of Tractor					
	All Median (IQR)		Open Median (IQR)		Filtered Median (IQR)	
Median × 10	12,480.43	(1465.16–51,033.02)	32,297.06	(3419.75–91,626.20)	7477.60	(1266.78–24,476.48)
Median × 100	54,768.74	(13,991.33–657,371.13)	91,626.20	N/A	17,911.28	(10,071.38–1,186,258.61)
Modified Z score	3798.88	(974.61–36,981.05)	32,297.06	(3419.75–94,427.33)	2474.77	(873.62–14,052.36)
Q3 + 1.5 IQR	2231.48	(462.77–25,104.17)	32,297.06	(16,382.42–61,961.63)	1823.82	(405.08–13,744.19)
Q3 + 2.5 IQR	12,355.15	(1823.82–61,961.63)	61,961.63	(47,129.34–76,793.91)	2231.48	(1416.15–12,355.15)

**Table 6:** Spearman correlation coefficients denoting correlation between the skin exposure extrapolated using the presented methods and 24-h using ethylene-bis-thiourea (ETU) levels.

Method	24-h Post-Exposure ETU Levels	24-h Post-Exposure ETU Level Corrected for Creatinine	Difference between 24-h Pre- and Post-Exposure ETU Levels
No outlier detection	0.46	0.46	0.39
Median × 10	0.68	0.65	0.49
Median × 100	0.50	0.47	0.37
Modified Z score	0.71	0.68	0.52
Q3 + 1.5 IQR	0.50	0.46	0.43
Q3 + 2.5 IQR	0.51	0.47	0.43

Spearman ρ values shown.

## References

[1] Mandic-Rajcevic S., Rubino F.M., Colosio C., Simeonov L., Macaev F., Simeonova B. (2013). General Approaches and Procedures for Pesticide Legislation. Environmental Security Assessment and Management of Obsolete Pesticides in Southeast Europe. NATO Science for Peace and Security Series C: Environmental Security.

[2] Colosio C., Rubino F.M., Alegakis A., Ariano E., Brambilla G., Mandic-Rajcevic S., Metruccio F., Minoia C., Moretto A., Somaruga C. (2012). Integration of biological monitoring, environmental monitoring and computational modelling into the interpretation of pesticide exposure data: Introduction to a proposed approach. Toxicol. Lett..

[3] Colosio C., Fustinoni S., Birindelli S., Bonomi I., De Paschale G., Mammone T., Tiramani M., Vercelli F., Visentin S., Maroni M. (2002). Ethylenethiourea in urine as an indicator of exposure to mancozeb in vineyard workers. Toxicol. Lett..

[4] Vitali M., Protano C., Del Monte A., Ensabella F., Guidotti M. (2009). Operative modalities and exposure to pesticides during open field treatments among a group of agricultural subcontractors. Arch. Environ. Contam. Toxicol..

[5] Aprea C., Terenzoni B., De Angelis V., Sciarra G., Lunghini L., Borzacchi G., Vasconi D., Fani D., Quercia A., Salvan A. (2004). Evaluation of skin and respiratory doses and urinary excretion of alkylphosphates in workers exposed to dimethoate during treatment of olive trees. Arch. Environ. Contam. Toxicol..

[6] Schneider T.A., Cherrie J.W., Vermeulen R., Kromhout H. (2000). Dermal exposure assessment. Ann. Occup. Hyg..

[7] OECD (1997). Guidance Document for the Conduct of Studies of Occupational Exposure to Pesticides During Agricultural Application.

[8] Fustinoni S., Mercadante R., Polledri E., Rubino F.M., Mandic-Rajcevic S., Vianello G., Colosio C., Moretto A. (2014). Biological monitoring of exposure to tebuconazole in winegrowers. J. Expo. Sci. Environ. Epidemiol..

[9] Mercadante R., Polledri E., Rubino F.M., Mandic-Rajcevic S., Vaiani A., Colosio C., Moretto A., Fustinoni S. (2018). Assessment of penconazole exposure in winegrowers using urinary biomarkers. Environ. Res..

[10] Rubino F.M., Mandic-Rajcevic S., Ariano E., Alegakis A., Bogni M., Brambilla G., De Paschale G., Firmi A., Minoia C., Micoli G. (2012). Farmers’ exposure to herbicides in North Italy: Assessment under real-life conditions in small-size rice and corn farms. Toxicol. Lett..

[11] Protano C., Guidotti M., Vitali M. (2009). Performance of different work clothing types for reducing skin exposure to pesticides during open field treatment. Bull. Environ. Contam. Toxicol..

[12] Mandic-Rajcevic S., Rubino F.M., Ariano E., Cottica D., Neri S., Colosio C. (2018). Environmental and biological monitoring for the identification of main exposure determinants in vineyard mancozeb applicators. J. Expo. Sci. Environ. Epidemiol..

[13] Hernández A.F., Tsatsakis A.M. (2017). Human exposure to chemical mixtures: Challenges for the integration of toxicology with epidemiology data in risk assessment. Food Chem. Toxicol..

[14] Tsatsakis A.M., Kouretas D., Tzatzarakis M.N., Stivaktakis P., Tsarouhas K., Golokhvast K.S., Rakitskii V.N., Tutelyan V.A., Hernandez A.F., Rezaee R. (2017). Simulating real-life exposures to uncover possible risks to human health: A proposed consensus for a novel methodological approach. Hum. Exp. Toxicol..

[15] Goumenou M., Tsatsakis A. (2019). Proposing new approaches for the risk characterisation of single chemicals and chemical mixtures: The Source Related Hazard Quotient (HQ_S_) and Hazard Index (HI_S_) and the Adversity Specific Hazard Index (HI_A_). Toxicol. Rep..

[16] Tsatsakis A., Goumenou M., Liesivuori J., Dekant W., Hernández A.F. (2019). Toxicology for real-life risk simulation—Editorial preface to this special issue. Toxicol. Lett..

[17] Kostoff R.N., Goumenou M., Tsatsakis A. (2018). The role of toxic stimuli combinations in determining safe exposure limits. Toxicol. Rep..

[18] Mandic-Rajcevic S., Rubino F.M., Vianello G., Fugnoli L., Polledri E., Mercadante R., Moretto A., Fustinoni S., Colosio C. (2015). Dermal exposure and risk assessment of tebuconazole applicators in vineyards. Med. Lav..

[19] Mandic-Rajcevic S., Rubino F.M., Ariano E., Cottica D., Negri S., Colosio C. (2019). Exposure duration and absorbed dose assessment in pesticide-exposed agricultural workers: Implications for risk assessment and modeling. Int. J. Hyg. Environ. Health.

[20] Colosio C., Visentin S., Birindelli S., Campo L., Fustinoni S., Mariani F., Tiramani M., Tommasini M., Brambilla G., Maroni M. (2006). Reference values for ethylenethiourea in urine in Northern Italy: Results of a pilot study. Toxicol. Lett..

[21] Shamsipour M., Farzadfar F., Gohari K., Parsaeian M., Amini H., Rabiei K., Hassanvand M.S., Navidi I., Fotouhi A., Naddafi K. (2014). A framework for exploration and cleaning of environmental data—Tehran air quality data experience. Arch. Iran. Med..

[22] O’Leary B., Reiners J.J., Xu X., Lemke L.D. (2016). Identification and influence of spatio-temporal outliers in urban air quality measurements. Sci. Total Environ..

[23] Yang J., Wang J., Zheng Y., Lei M., Yang J., Wan X., Chen T. (2018). Method for identifying outliers of soil heavy metal data. Environ. Sci. Pollut. Res. Int..

[24] Durkin P.R., Rubin L., Withey J., Meylan W. (1995). Methods of assessing dermal absorption with emphasis on uptake from contaminated vegetation. Toxicol. Ind. Health.

[25] Grover R., Franklin C.A., Muir N.I., Cessna A.J., Riedel D. (1986). Dermal exposure and urinary metabolite excretion in farmers repeatedly exposed to 2,4-D amine. Toxicol. Lett..

[26] Fargnoli M., Lombardi M., Puri D., Casorri L., Masciarelli E., Mandić-Rajčević S., Colosio C. (2019). The Safe Use of Pesticides: A Risk Assessment Procedure for the Enhancement of Occupational Health and Safety (OH_S_) Management. Int. J. Environ. Res. Public Health.

[27] Mosteller R.D. (1987). Simplified calculation of body-surface area. N. Engl. J. Med..

[28] Moore R.A., Waheed A., Burns B. (2019). Rule of Nines.

[29] Jones K., Patel K., Cocker J., Bevan R., Levy L. (2010). Determination of ethylenethiourea in urine by liquid chromatography-atmospheric pressure chemical ionisation-mass spectrometry for monitoring background levels in the general population. J. Chromatogr. B. Analyt. Technol. Biomed. Life Sci..

[30] R Core Team (2017). R: A Language and Environment for Statistical Computing.

[31] Iglewicz B., Hoaglin D.C. (1993). How to Detect and Handle Outliers.

[32] De Cock J., Heederik D., Boleij J.S.M., Kromhout H., Hoek F., Wegh H., Ny E.T. (1998). Exposure to Captan in Fruit Growing. Am. Ind. Hyg. Assoc. J..

[33] Hines C.J., Deddens J.A., Coble J., Kamel F., Alavanja M.C.R. (2011). Determinants of captan air and dermal exposures among orchard pesticide applicators in the agricultural health study. Ann. Occup. Hyg..

[34] Coble J., Arbuckle T., Lee W., Alavanja M., Dosemeci M. (2005). The validation of a pesticide exposure algorithm using biological monitoring results. J. Occup. Environ. Hyg..

[35] Yoshida K., Fuzesi I., Suzan M., Nagy L. (1990). Measurements of surface contamination of spray equipment with pesticides after various methods of application. J. Environ. Sci. Health Part B.

[36] Sanco E. (2009). Review Report for the Active Substance Mancozeb.

[37] Kennedy M.C., Glass C.R., Fustinoni S., Moretto A., Mandic-Rajcevic S., Riso P., Turrini A., van der Voet H., Hetmanski M.T., Fussell R.J. (2015). Testing a cumulative and aggregate exposure model using biomonitoring studies and dietary records for Italian vineyard spray operators. Food Chem. Toxicol..

[38] Brouwer M., Huss A., Vermeulen R., Nijssen P., De Snoo G., Kromhout H. (2014). Expert assessment of historical crop specific pesticide use in the Netherlands. Occup. Environ. Med..

[39] Acquavella J.F., Alexander B.H., Mandel J.S., Burns C.J., Gustin C. (2006). Exposure misclassification in studies of agricultural pesticides: Insights from biomonitoring. Epidemiology.

[40] Aprea C., Betta A., Catenacci G., Colli A., Lotti A., Minoia C., Olivieri P., Passini V., Pavan I., Roggi C. (1997). Urinary excretion of ethylenethiourea in five volunteers on a controlled diet (multicentric study). Sci. Total Environ..

